# Sodium Fumarate Enhances the Antimicrobial Efficacy of a Commercial Acidic Disinfectant Against *Listeria monocytogenes*, *Escherichia coli* and *Salmonella* Typhimurium Inoculated on Fresh Produce

**DOI:** 10.3390/foods15132339

**Published:** 2026-07-02

**Authors:** Ruth H. Barnes, Charlotte Delattre, Tolulope Olowomoffe, Konstantina Kourmentza, Kimon Andreas G. Karatzas

**Affiliations:** 1 Department of Food and Nutritional Sciences, University of Reading, Whiteknights, Reading RG6 6AP, UK; 2 Ecole de Biologie Industrielle, 49 Avenue des Genottes, 95800 Cergy, France; 3 Department of Chemical and Environmental Engineering, University of Nottingham, University Park, Nottingham NG7 2RD, UK

**Keywords:** food safety, fruits, foodborne pathogens, fumaric acid, organic acids, decontamination

## Abstract

This study investigated the efficacy of sodium fumarate combined with the commercial organic acid disinfectant NatureSeal FS (FS) against the prominent foodborne pathogens *Listeria monocytogenes*, *Escherichia coli* and *Salmonella* Typhimurium. Sodium fumarate at 10 mM enhanced the antimicrobial activity of FS against overnight cultures of all three pathogens in growth media within 1–5 min. Subsequently, FS supplemented with 25 mM sodium fumarate (pH 2.4) showed enhanced antimicrobial activity by 1–2 log cycles, reaching a total of 2.14–3.22 log cycles within 5 min against the three pathogens inoculated each one individually, on the surface of strawberries, pears and apples compared to a <1.1 log reduction for all control treatments (no treatment, water, 100 ppm chlorine and FS) at pH 2.4. Then, six different organic acid mixes containing key components of FS, two of which were supplemented with 50 and 25 mM sodium fumarate, were tested against the three pathogens, which also performed significantly better than the rest. Sodium fumarate enhanced the efficacy of a commercial acidic disinfectant on fresh produce significantly. The results of this study are highly important for the food industry and consumer protection, as the use of sodium fumarate could significantly enhance the food safety of fresh produce, which is the main contributor to foodborne illness nowadays.

## 1. Introduction

In the past, fresh fruits and vegetables were considered relatively low risk in terms of food safety. However, the occurrence of foodborne illnesses caused by pathogenic organisms such as *Escherichia coli* strains, *Salmonella* and *Listeria monocytogenes*, associated with fresh produce, appears to increase rapidly [1,2]. Several studies now suggest that up to 46% of all foodborne illness is associated with fruits, nuts and vegetables [3,4]. The main reasons behind this are the increase in global consumption of fresh produce, its wider distribution, resulting in more widespread incidents, and the increasingly complex food chains leading to traceability and control issues, combined with poor production and handling techniques [1,5,6,7,8,9].

There is currently a wide range of treatments and technologies designed to help reduce the levels of foodborne pathogens. The most common treatments used worldwide include washes or dips. These solutions may include sodium hypochlorite, organic acids (such as citric or acetic), hydrogen peroxide, peroxyacetic acid, calcium hypochlorite, ozone and electrolysed water [8,9,10,11,12,13,14]. Despite their wide use, they have a limited effectiveness, which is normally approximately 1 log reduction as determined by a comprehensive analysis of the antimicrobial effects of various disinfectants on numerous types of fruits and vegetables [1,15].

Chlorine-based disinfectants have been popular with producers as effective and inexpensive sanitisers. However, recently, there has been a concern amongst consumers that these may have adverse carcinogenic effects, forcing producers to look for more acceptable alternatives [16]. Such alternatives include organic acids, which are already used by the food industry as preservatives and flavour enhancers, i.e., citric acid and acetic acid [1,16]. These weak acids are “Generally Recognised as Safe” (GRAS), and easily acceptable by consumers and producers [16]. Therefore, they are used individually or in combination to treat fresh produce [9].

Organic acid treatments such as citric acid, acetic acid, tartaric acid and malic acid have been found to have significant antimicrobial action [13,16]. The mode of action of these weak organic acids is based on their appearance in their dissociated and undissociated form. The undissociated form of a weak acid can passively diffuse through the bacterial membrane and enter the cytoplasm [17]. As the intracellular pH of many organisms is normally close to neutral, further dissociation of the undissociated molecules that enter the cell takes place, releasing more protons intracellularly, leading to further cellular damage. The acid may also interfere with membrane transport and permeability, metabolic pathways, result in an increase of the osmotic pressure and eventually cause bacterial death [16,17,18].

An example of such a commercial organic acid treatment, referred here as FS, comprises a combination of organic acids, resulting in a low pH solution. This is normally used to wash fresh produce at a pH range between 2.4 and 2.8 as recommended by its producer [19]. It has been shown that some organic acids may have a different level of antimicrobial activity from that predicted by the classical model, considering only the dissociation constant of the specific acid. For example, it has been previously reported that fumaric acid can interact with key mechanisms of acid resistance of certain foodborne pathogens, possibly resulting in an increased antimicrobial action under acidic conditions [20]. Compounds such as maleic or fumaric acid can negatively impactamino acid decarboxylase systems (e.g., GAD system) and possibly other mechanisms, enhancing the sensitivity of pathogens and spoilage organisms [20,21]. This is considered advantageous compared to other acids, such as acetic and lactic acid [22,23] and could increase the efficacy of disinfection or allow the use of higher pH values or a shorter time, limiting the damage to the fresh produce.

Fumaric acid is a dicarboxylic organic acid, used in the food industry as an acidity regulator. It is effective against several different organisms, including *Escherichia coli* O157:H7, *L. monocytogenes*, and *Salmonella.* Its effectiveness has been demonstrated for a number of food products, including fresh produce [23,24,25]. In previous work, we have demonstrated that fumarate, despite its low predicted antimicrobial activity based on its low dissociation constant (pKa_1_: ≈3.03 and pKa_2_: ≈4.5), is highly antimicrobial towards planktonic cells of *L*. *monocytogenes* and *E. coli* and biofilms of *L. monocytogenes* mainly due to its negative effect on the glutamate decarboxylase (GAD) system of the above bacteria [20]. As such, fumarate could be used to enhance the efficacy of acidic disinfectants against pathogenic and spoilage bacteria on fresh produce and thus contribute in the reduction of foodborne illness and spoilage.

However, fumaric acid has a low solubility in aqueous solutions. To resolve this problem, its salts, including sodium fumarate, may be employed as alternatives [26]. Salts of fumaric acid and more specifically those with sodium are used worldwide in food applications, while in most cases, there is no maximum limit. Furthermore, fumarate is present in most living organisms, as it is part of the TCA cycle and therefore, it is found in most foods. Currently, the US Food and Drug Administration (FDA) and Food and Agriculture Organisation (FAO) have approved fumaric acid and its salts (e.g., calcium, sodium, etc.) for use as generally recognised as safe (GRAS) additives in foods [27,28], while they are also used in Canada, Australia, New Zealand and many other parts of the world. In the European Union (EU), fumaric acid (E297) is an acceptable food additive with a maximum limit of 4000 mg/L (34.46 mM) with voluntary enforcement [29]. Fumaric acid is currently under re-evaluation by the European Food & Safety Authority (EFSA) [30] while sodium fumarate (E365) is not an acceptable food additive [31]. However, in the present work, sodium fumarate is not being investigated as a food additive but as a part of a disinfection regime.

The aim of our study was to examine the potential use of very low concentrations of sodium fumarate (25–50 mM) in a disinfection regime of fresh produce. These concentrations were equal to or below the equivalent concentration that is acceptable for fumaric acid in the EU. Therefore, this work could be highly beneficial for disinfection of fresh produce and reduction in foodborne illness and food spoilage around the world and in the EU.

## 2. Materials and Methods

### 2.1. Bacterial Strains and Growth Conditions

All bacterial strains used (Table 1) were revived from 2 mL cryovials stored at −80 °C cryopreserved with the addition of 7% dimethyl sulfoxide (DMSO; Sigma-Aldrich, Gillingham, Dorset, UK). *L. monocytogenes* LO28 is a typed strain that has been previously used in acid stress work [32,33,34], and it was grown onto Tryptic Soy Broth Agar (Oxoid, Basingstoke, UK) supplemented with 0.5% yeast extract (TSBY agar; Oxoid, Basingstoke, UK). Both *E. coli* O157:H7, a non-verocytotoxic strain (missing *stx1* or *stx2*), and *S.* Typhimurium 30 (from bovine source) have also been previously used in acid stress work [35], and were both grown on Lysogeny Broth (LB; LABM, Heywood, Lancashire, UK). Three colonies from each agar plate were taken with an inoculation loop and transferred into TSBY broth (Oxoid, Basingstoke, UK) for *L. monocytogenes* LO28, and LB broth (LABM, Heywood, Lancashire, UK) for *E. coli* O157:H7 and *S.* Typhimurium 30 in 10 mL Bijou bottles. These overnight cultures were incubated overnight at 37 °C and used to inoculate 250 mL Erlenmeyer flasks (1% inoculum), containing similar media as the first overnight. Subsequently, these experimental cultures were incubated overnight at 37 °C with shaking at 150 rpm for 18 h.

### 2.2. Determination of Minimum Inhibitory Concentration (MIC)

Concentrations ranging from 0 to 200 mM tartaric acid, citric acid, malic acid and sodium fumarate (Sigma-Aldrich, Dorset, UK) were mixed with an 1% solution of an overnight culture of either *L. monocytogenes* LO28, *E. coli* O157:H7 or *S.* Typhimurium 30. Due to the limited solubility of fumaric acid, lower concentrations ranging from 0 to 34 mM were prepared and inoculated in the same way. Cultures were inoculated into 96-well plates and incubated at 37 °C with shaking. The optical density of the cultures was assessed at 620 nm (OD_620nm_) after 24 h using a Tecan Sunrise absorbance microplate reader (Tecan Group AG, Männedorf, Switzerland) and a Magellan™ data analysis software version 7.2 to define the MIC in each case. Experiments were performed in three independent biological replicates, where cells used in each replicate were derived from a different set of 3 colonies grown separately. For each biological replicate, measurements were taken in three technical replicates.

### 2.3. Survival Under Severe Acidic Conditions in the Presence of Sodium Fumarate

Experimental cultures of 20 mL were prepared in TSBY for *L. monocytogenes* LO28 and LB for *E. coli* O157:H7 and *S.* Typhimurium 30 as described above. Subsequently, cultures were further grown in 250 mL Erlenmeyer flasks at 37 °C under agitation at 150 rpm. Acid challenges were performed using an organic acid mix disinfectant FS (Agricoat NatureSeal; Hungerford, Berkshire, UK) in the presence or absence of 10 mM sodium fumarate at pH 2.4 for *L. monocytogenes* LO28 and *E. coli* O157:H7. For *S.* Typhimurium 30, a pH of 2.8 was used as the pH 2.4 caused a rapid inactivation. The pH was adjusted through the addition of FS, as recommended by the disinfectant producer [19]. One hundred μL samples were taken at various time points for *L. monocytogenes* LO28 and *S.* Typhimurium 30 until 120 s and until 600 s for *E. coli* O157:H7. All samples were then placed in 900 μL maximum recovery diluent (MRD; Oxoid Limited, Hampshire, UK). Ten-fold serial dilutions were prepared, and 10 μL of each dilution was plated onto BHI agar plates and incubated at 37 °C for 24 h. Colonies were then counted to assess the concentration of cells in the culture at each time point. Experiments were performed in three independent biological replicates, where cells used in each replicate were derived from a different set of 3 colonies grown separately. For each biological replicate, one series of decimal dilutions was performed, which, however, was spot-plated in triplicate, comprising 3 technical replicates for each biological replicate.

### 2.4. Comparison of Treatments on Fresh Produce

To examine the effect of different treatments on fresh produce, three types of fruit were selected: (a) apples (Granny Smith variety), (b) pears (Conference variety) and (c) strawberries (Sweet Eve variety). All fruits were washed with deionised water and were left to dry. Following this, 10 g samples of each fruit were cut with a sterile scalpel and weighed out under sterile conditions. To ensure a similar surface area, similar-sized fruits were used, while they were all sliced in a similar way. Subsequently, the samples were washed with 70% ethanol to inactivate any residual bacteria. Samples were allowed to air dry in a laminar flow hood for 1 h. Each sample was then inoculated with 100 μL of an overnight culture of either *E. coli* O157:H7, *L. monocytogenes* LO28 or *S.* Typhimurium 30, prepared as previously described. Samples were then placed in Petri dishes and stored at 4 °C for 24 h to allow for bacterial attachment and to mimic conditions under which such produce might be stored or transported.

The following four treatment solutions were prepared: (a) deionised water, (b) deionised water with 100 ppm free chlorine, prepared using calcium hypochlorite, (c) FS at pH 2.4 and (d) FS at pH 2.4 supplemented with 25 mM sodium fumarate. Each sample of inoculated fruit was submerged in 50 mL of each of these treatment solutions for 5 min at room temperature. Samples were then transferred to a stomacher bag containing 90 mL MRD, and this decimal dilution resulted in the immediate inactivation of the disinfectants, especially that of the acidic disinfectants, since the pH was immediately raised far above their activity range. This was assessed in separate experiments (data not shown). Subsequently, the samples were left for 1 min in a Colworth stomacher 400 (Seaward, Durham, UK) in order to be homogenised, and immediately, ten-fold serial dilutions were prepared (100 μL in 900 μL) in MRD. Subsequently, 10 μL of each dilution was plated onto LB agar for *E. coli* O157:H7 and *S.* Typhimurium 30 and TSBY agar for *L. monocytogenes* LO28 (LABM, Lancashire, UK). All plates were incubated at 37 °C for 24 h, and subsequently, colonies were counted to assess the concentration of viable cells for each sample. Experiments were performed in three independent biological replicates, where cells used in each replicate were derived from a different set of 3 colonies grown separately. For each biological replicate, one series of decimal dilutions was performed, which was, however, spot-plated in triplicate, comprising 3 technical replicates for each biological replicate.

### 2.5. Survival of E. coli O157:H7, L. monocytogenes LO28 and S. Typhimurium 30 Against Reformulated FS Organic Acid Treatments

Cultures of *E. coli* O157:H7, *L. monocytogenes* LO28 and *S.* Typhimurium 30 were prepared using the protocol described above (Section 2.1). All samples were transferred into 50 mL Falcon^®^ tubes (VWR, Leicestershire, UK). Samples were then centrifuged (Eppendorf, Hamburg, Germany) at 12,000× *g* for 10 min. The supernatant was then discarded, and the pellet was dispersed using an inoculation loop. Subsequently, 20 mL of either FS (pH 2.8), HCL (pH 2.8) or one of the organic acid treatment solutions described in Table 2 (pH 2.8) was added to the sample and vortexed for 10 s. For these experiments, we examined the worst-case scenario and used the mildest acidic treatment, at the highest pH limit of what is recommended for FS (pH range: 2.4–2.8).

Subsequently, 100 μL samples of *E. coli* O157:H7 and *L. monocytogenes* LO28 were obtained at 0, 5, 10 and 20 min. Samples of *S.* Typhimurium 30 were obtained at 0, 30, 60 and 120 s. The 100 μL samples were then placed in 900 μL of MRD. Ten-fold serial dilutions were prepared, and 10 μL of each dilution was plated onto LB agar (LABM, Lancashire, UK) for *E. coli* O157:H7 and *S.* Typhimurium 30 or TSBY agar for *L. monocytogenes* LO28, and all plates were incubated at 37 °C for 24 h. All the above experiments were performed at room temperature. The selection of room temperature was based on the worst-case scenario where low temperature is not maintained well, as these treatments could often take place in an open space and warm external temperatures, while it is known that higher temperatures enhance acid resistance [40]. Experiments were performed in three independent biological replicates, where cells used in each replicate were derived from a different set of 3 colonies grown separately. For each biological replicate, one series of decimal dilutions was performed, which, however, was spot-plated in triplicate, comprising 3 technical replicates for each biological replicate.

### 2.6. Statistical Analysis

In all cases, experiments were run in independent biological triplicates (*n* = 3) where cells used in each replicate were derived from a different set of 3 colonies grown separately. As mentioned above, for each biological replicate, one series of decimal dilutions was performed, which, however, was spot-plated in triplicate, comprising 3 technical replicates for each biological replicate. Values are presented as the average of the biological replicates with standard deviation (SD).

**Figure 1:**
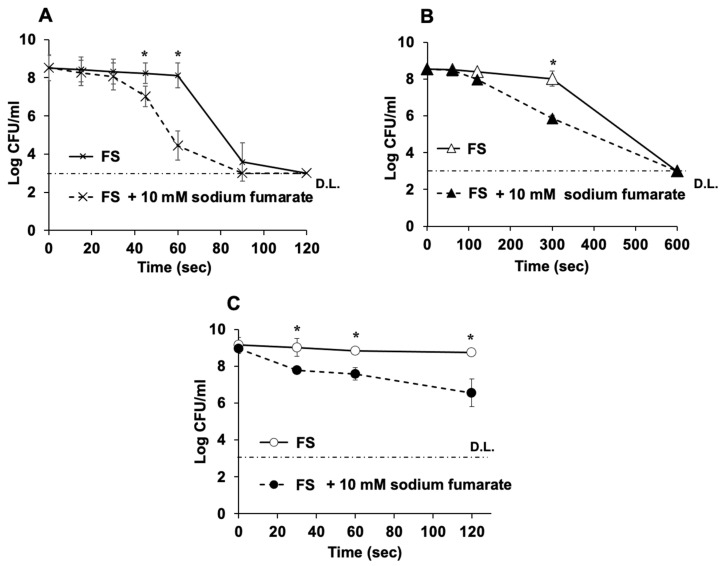
Survival of *L. monocytogenes* LO28 (**A**) in the presence of FS alone (x) or FS supplemented with 10 mM sodium fumarate (X) and of *E. coli* O157:H7 (**B**) in the presence of FS alone (△) or FS supplemented with 10 mM sodium fumarate (▲) at pH 2.4. Survival of *S.* Typhimurium 30 (**C**) in the presence of FS alone (◯) or FS supplemented with 10 mM sodium fumarate (●) at pH 2.8. Asterisks denote statistical significance using a paired Student’s *t*-test (*p* < 0.05), and D.L denotes the detection limit of the experimental setup. Asterisks (*) denote statistically significant difference between the FS + 10 mM sodium fumarate and the control FS.

For survival experiments presented in Figure 1, results were assessed using a paired Student’s *t*-test. A *p*-value of <0.05 denoted statistically significant results, which have been indicated by an asterisk in the relevant subfigures.

**Figure 2:**
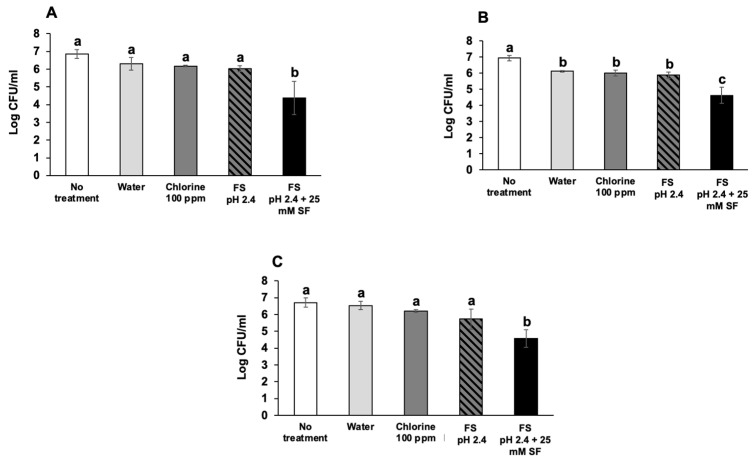
Survival of *L. monocytogenes* LO28 (**A**), *E. coli* O157:H7 (**B**) and *S.* Typhimurium 30 (**C**) inoculated onto the surface of strawberries using treatments with water, 100 ppm chlorine, FS (pH 2.4) and FS supplemented with 25 mM sodium fumarate (pH 2.4). Bars represent average values of independent biological triplicate trials (*n* = 3), and error bars represent the standard deviation. One-way analysis of variance (ANOVA) was performed to identify variations within each panel, and lowercase letters designate post hoc statistical partitions resolved via Tukey’s HSD test evaluated independently (*p* < 0.05). Treatments sharing a common letter within a panel possess no statistically significant differences, whereas entirely separate letter classifications indicate significant divergence in performance.

**Figure 3:**
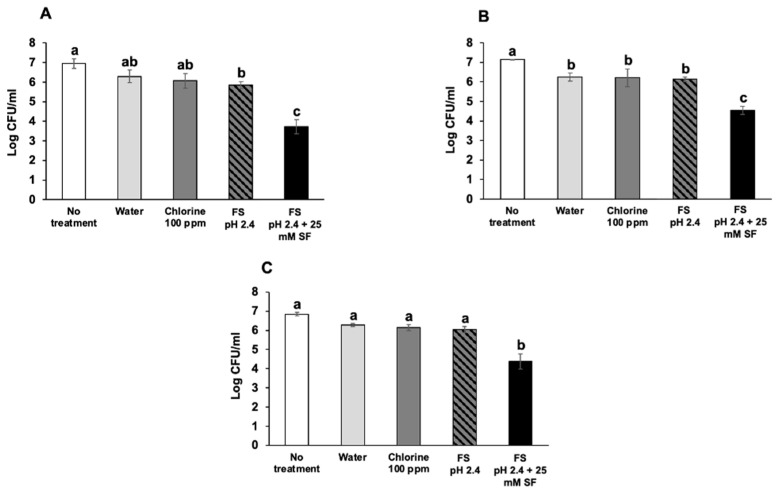
Survival of *L. monocytogenes* LO28 (**A**), *E. coli* O157:H7 (**B**) and *S.* Typhimurium 30 (**C**) inoculated onto the surface of pears using treatments with water, 100 ppm chlorine, FS organic acid treatment (pH 2.4) and FS supplemented with 25 mM sodium fumarate (pH 2.4). Bars represent average values of independent biological triplicate trials (*n* = 3), and error bars represent the standard deviation. One-way analysis of variance (ANOVA) was performed to identify variations within each panel, and lowercase letters designate post hoc statistical partitions resolved via Tukey’s HSD test evaluated independently (*p* < 0.05). Treatments sharing a common letter within a panel possess no statistically significant differences, whereas entirely separate letter classifications indicate significant divergence in performance.

**Figure 4:**
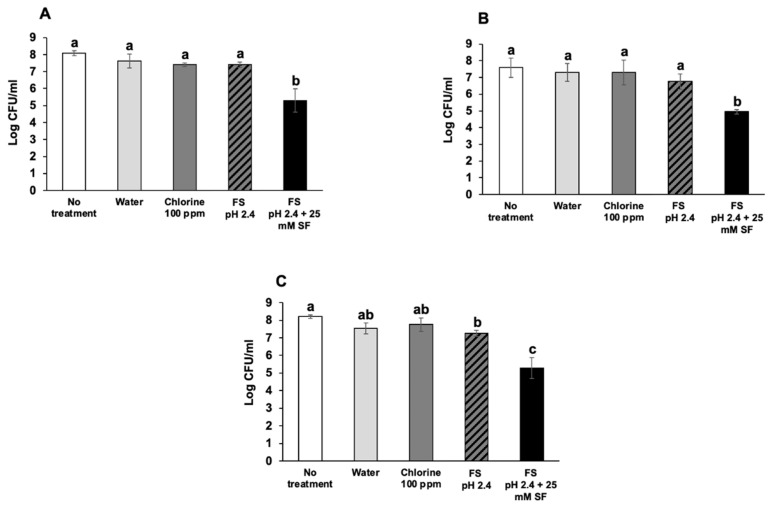
Survival of *L. monocytogenes* LO28 (**A**), *E. coli* O157:H7 (**B**) and *S.* Typhimurium 30 (**C**) inoculated onto the surface of apples using treatments with water, 100 ppm chlorine, FS (pH 2.4) and FS supplemented with 25 mM sodium fumarate (pH 2.4). Bars represent average values of independent biological triplicate trials (*n* = 3), and error bars represent the standard deviation. One-way analysis of variance (ANOVA) was performed to identify variations within each panel, and lowercase letters designate post hoc statistical partitions resolved via Tukey’s HSD test evaluated independently (*p* < 0.05). Treatments sharing a common letter within a panel possess no statistically significant differences, whereas entirely separate letter classifications indicate significant divergence in performance.

**Figure 5:**
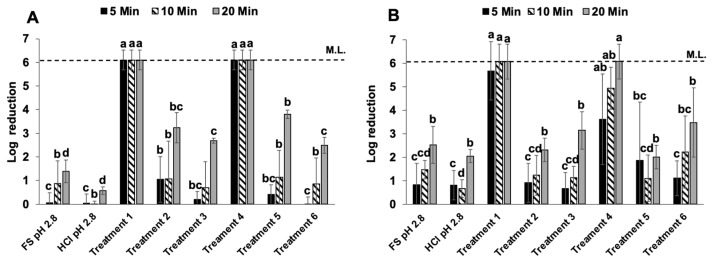
Cultures challenged with FS treatment, HCL and six reformulated treatments based on the original FS l. *L. monocytogenes* LO28 sampled at 0, 5, 10 and 20 min (**A**), *E. coli* O157:H7 sampled at 0, 5, 10 and 20 min (**B**). Bars represent average values of independent biological triplicate trials (*n* = 3), and error bars represent the standard deviation. One-way analysis of variance (ANOVA) was performed to identify variations within each panel, and lowercase letters designate post hoc statistical partitions resolved via Tukey’s HSD test evaluated independently (*p* < 0.05). Treatments sharing a common letter within a panel possess no statistically significant differences, whereas entirely separate letter classifications indicate significant divergence in performance.

**Figure 6:**
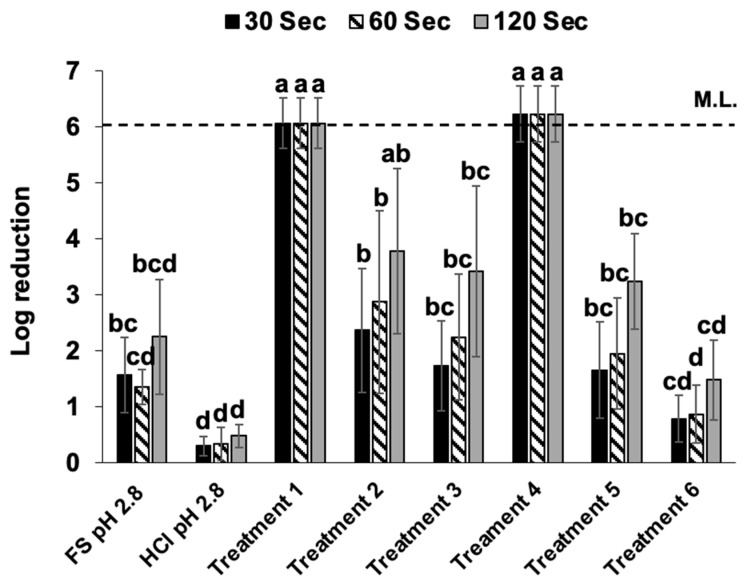
Survival of *S*. Typhimurium 30 following challenge with FS treatment, HCL and six reformulated treatments based on the original FS, sampled at 0, 30, 60 and 120 s. Bars represent average values of independent biological triplicate trials (*n* = 3), and error bars represent the standard deviation. One-way analysis of variance (ANOVA) was performed to identify variations, and lowercase letters designate post hoc statistical partitions resolved via Tukey’s HSD test evaluated independently (*p* < 0.05). Treatments sharing a common letter possess no statistically significant differences, whereas entirely separate letter classifications indicate significant divergence in performance.

For all other experiments (Figure 2, Figure 3, Figure 4, Figure 5 and Figure 6) to evaluate the variations among the treatment groups, data within each independent panel were analysed separately using an One-Way Analysis of Variance (ANOVA). Pairwise multiple comparisons of the group averages within each panel were performed using Tukey’s Honestly Significant Difference (HSD) post hoc test to control the experiment-wise Type I error rate. For all tests, statistical significance was defined a priori using a threshold of 0.05 (*p* < 0.05).

## 3. Results

### 3.1. Growth in the Presence of Selected Acids

The MICs of various acids used in the work against 3 pathogenic microorganisms were assessed (Table 3). Overall, apart from sodium fumarate, the MICs of the different compounds ranged between 12.5 and 50 mM, with no major differences between the different compounds, while the MICs against *L. monocytogenes* LO28 were higher. The MIC of fumaric acid was similar (34 mM) for all microorganisms tested. It should be noted that although we were able to determine the MIC of fumaric acid, its concentration range was limited as its maximum solubility in aqueous solutions is 0.7% *w*/*v* (60 mM) [41]. Due to this, a different range of concentrations was tested for this compound.

Sodium fumarate did not show an MIC for the range of the concentrations tested, and no inhibition was observed as expected, since it requires acidic conditions to exert its antimicrobial effects. Under the conditions of the experiment, the pH was not reduced since sodium fumarate is a salt, and it does not affect the pH.

### 3.2. Survival After Exposure to a Commercial Acidic Disinfectant in the Presence of Sodium Fumarate

For *L. monocytogenes* LO28, the supplementation of FS with 10 mM sodium fumarate resulted in a 4.1 log reduction within 60 s, compared to 0.20 log reduction elicited by FS alone at pH 2.4 (additional 3.9 log reduction; *p* < 0.05; Figure 1A). Similar results were obtained with *L. monocytogenes* 10403S, where supplementation of FS with 10 mM sodium fumarate resulted in a statistically significant >5 log higher inactivation than FS alone at pH 2.4 and 2.8 within 120 and 360 s, respectively (Figure S1A,B; Supplementary Data).

The addition of 10 mM sodium fumarate resulted in a 2.57 log reduction in *E. coli* O157:H7 compared to 0.51 for FS alone after 300 s (additional 2.06 log reduction; *p* < 0.05; Figure 1B). We also obtained similar results with *E. coli* K12 (grown in LB), where the addition of FS and 10 mM sodium fumarate resulted in a statistically significant 3.5 log cycles higher inactivation than FS alone at pH 2.4 after 30 s (Figure S2; Supplementary Data).

Similarly to the other bacteria, the presence of both FS and 10 mM sodium fumarate elicited a 2.4 log reduction on *S.* Typhimurium 30 (grown in LB) compared to 0.4 for FS alone following 120 s of treatment at pH 2.8 (additional 2 log cycle reduction; *p* < 0.05; Figure 1C).

### 3.3. Survival of E. coli O157:H7, L. monocytogenes LO28 and S. Typhimurium 30 on Fresh Produce When Exposed to Various Aqueous Treatments

#### 3.3.1. Disinfection of Strawberries

In these experiments, we assessed different treatments on strawberries that were inoculated with the three different foodborne pathogens used in this study, including a 5 min treatment with water, 100 ppm chlorine, FS at pH 2.8 and FS at pH 2.8 supplemented with 25 mM sodium fumarate.

Regarding *L. monocytogenes* LO28, 5 min treatments with water, 100 ppm chlorine and FS at pH 2.4 resulted in minor log reductions of 0.55, 0.67 and 0.83, respectively, which were not statistically different from the non-treated control. Interestingly, FS supplemented with 25 mM sodium fumarate (pH 2.4) resulted in a statistically significant 2.4 log reduction compared to the control (no treatment) or treatment with only water (*p* < 0.05). The combined treatment was the most successful, and it was far more effective than chlorine and FS by an additional 1.7 and 1.6 log reduction, respectively (Figure 2A).

For *E. coli* O157:H7, the 5 min treatments of water, 100 ppm chlorine and FS at pH 2.4 resulted in minor log reductions of 0.78, 0.89 and 1.03, respectively (Figure 2B). The latter treatments resulted in statistically significant, but showed minor reductions compared to the untreated control (*p* < 0.05). Similarly, to *L. monocytogenes*, with *E. coli* O157:H7 supplementation of FS (pH 2.4) with 25 mM sodium fumarate resulted in a higher inactivation than all other treatments with statistical significance (*p* < 0.05).

This inactivation was 2.28 log higher than the non-treated control and the water treatment (*p* < 0.05). Also, supplementation of FS with fumarate resulted in a 1.25 and 1.4 log cycles higher reduction than FS or chlorine alone, respectively (Figure 2B).

In the case of *S.* Typhimurium 30, the 5 min treatments of water, 100 ppm chlorine and FS at pH 2.4, resulted in minor log reductions of 0.17, 0.51 and 0.97, respectively, without any statistical significance when compared to the control (no treatment; Figure 2C). Supplementation of FS with 25 mM sodium fumarate (pH 2.4, 5 min treatment) resulted in a statistically significant 2.14 log reduction compared to all other treatments. The combined treatment had an improved efficacy of an additional 1.63 and 1.16 log reduction over that of chlorine and FS, respectively (Figure 2C).

#### 3.3.2. Disinfection of Pears

In the experiments with pears, similarly to experiments with strawberries, the combined treatment of FS and sodium fumarate was the most effective (Figure 3). In the case of *L. monocytogenes* LO28, 5 min treatments with water, 100 ppm chlorine and FS at pH 2.4, resulted in minor log reductions of 0.65, 0.87 and 1.10, respectively, with the latter being statistically more effective compared to the untreated control (*p* < 0.05). The combined treatment of FS and 25 mM sodium fumarate (pH 2.4; 5 min) resulted in a 3.21 log reduction, which was statistically higher than all other treatments (*p* < 0.05). The latter treatment was the most successful, and it was significantly more effective than chlorine and FS by an additional 2.30 and 2.11 log reduction, respectively (Figure 3A).

With *E. coli* O157:H7, the 5 min treatments of water, 100 ppm chlorine and FS at pH 2.4, resulted in minor log reductions in CFU/mL of 0.89, 0.94 and 1.01, respectively (Figure 3B). All these reductions were statistically higher than the control (no treatment) (*p* < 0.05). Once more, the combined treatment of FS and 25 mM sodium fumarate resulted in a log reduction of 2.61, which was statistically more effective than all other treatments (*p* < 0.05). The combined treatment was more effective by an additional 1.67 and 1.60 log reduction over chlorine and FS, respectively (Figure 3B).

In the case of *S.* Typhimurium 30, the 5 min treatments of water, 100 ppm chlorine and FS at pH 2.4, resulted in minor and not statistically significant log reductions of 0.56, 0.68 and 0.79, respectively, compared to the untreated control (Figure 3C). Also in this case, the 5 min treatment of FS with 25 mM sodium fumarate (pH 2.4) resulted in a log reduction of 2.45, which was statistically more effective than all other treatments (*p* < 0.05). The combined treatment resulted in an additional 1.77 and 1.66 log reduction over that of chlorine and FS, respectively (Figure 3C).

#### 3.3.3. Disinfection of Apples

With apples, the combined treatment of FS and sodium fumarate was the most effective (Figure 4). When *L. monocytogenes* LO28 was used, 5 min treatments with water, 100 ppm chlorine and FS at pH 2.4 resulted in minor log reductions of 0.45, 0.68 and 0.67, respectively, which were not statistically different from the untreated control (*p* < 0.05). Only the combined treatment of FS and 25 mM sodium fumarate (pH 2.4; 5 min) resulted in a 2.79 log reduction, which was more effective than all others with statistical significance (*p* < 0.05). The latter treatment was the most antimicrobial, and it was significantly more effective than chlorine and FS by an additional 2.11 and 2.12 log reduction, respectively (Figure 4A).

With *E. coli* O157:H7, the 5 min treatments of water, 100 ppm chlorine and FS at pH 2.4, resulted in minor and not statistically significant log reductions of 0.28, 0.28 and 0.84, respectively (Figure 4B). Once more, the combined treatment of FS and 25 mM sodium fumarate resulted in a 2.64 log reduction that was statistically more effective than all other treatments (*p* < 0.05). This treatment was more effective by an additional 2.36 and 1.80 log reduction over that of chlorine and FS, respectively (Figure 4B).

In the case of *S.* Typhimurium 30, the 5 min treatments of water, 100 ppm chlorine and FS at pH 2.4, resulted in minor log reductions of 0.67, 0.45 and 0.94, respectively (Figure 4C). From the above, only the FS treatment was statistically more effective than the untreated control (*p* < 0.05). The 5 min treatment of FS with 25 mM sodium fumarate (pH 2.4) resulted in a log reduction of 2.93 that was statistically more effective than all others (*p* < 0.05). The combined treatment was more effective by an additional 2.47 and 1.98 log reduction over that of chlorine and FS, respectively (Figure 4C).

### 3.4. Survival of L. monocytogenes LO28, E. coli O157:H7 and S. Typhimurium 30 Against Various Reformulated FS Organic Acid Treatments

In total, six alternative treatments were examined for their bactericidal efficacy against *L. monocytogenes* LO28, *E. coli* O157:H7 and *S.* Typhimurium 30. These combinations were based on variations in the composition of the FS (pH 2.8) with alterations in the proportion of its key constituent acids (malic, tartaric, citric and trisodium citrate) or with the addition of sodium fumarate in two cases (Table 2).

In the case of *L. monocytogenes* LO28, FS treatment (pH 2.8) and HCL (pH 2.8) resulted in minor but significant log reductions of CFU/mL of 1.38 and 0.57 after 20 min. The six reformulated treatments 1, 2, 3, 4, 5 and 6 that were based on the original FS (pH 2.8) elicited larger log reductions of 6.10, 3.23, 2.68, 6.10, 3.80 and 2.48, respectively, and they were all found to confer a significant improvement over the original FS treatment after 20 min (*p* < 0.05; Figure 5A). Treatments containing sodium fumarate (1 and 4) resulted in the highest and most rapid inactivation. Inactivation in the presence of sodium fumarate was highly effective since, within 5 min, the numbers of *L. monocytogenes* dropped below the detection limit (>6.1 log reduction of CFU/mL), while all other treatments showed a lower than 1 log reduction at that time point (Figure 5A). Due to the centrifugation of the overnight cultures used, the detection limit varied slightly between replicates and samples, and this is why we reported the detection limit as >6.1.

In the case of *E. coli* O157:H7, FS treatment (pH 2.8) and HCL (pH 2.8) resulted in significant log reductions of 2.52 and 2.05 after 20 min. The six reformulated treatments 1, 2, 3, 4, 5 and 6 that were based on the original FS (pH 2.8) resulted in 6.07, 2.31, 3.14, 6.07, 2.00 and 3.48 log reduction, respectively. Two of the reformulated treatments (1 and 4) were found to offer a significant improvement over the original FS treatment (pH 2.8) after 20 min (*p* < 0.05; Figure 5B). Similarly to *L. monocytogenes* LO28, with *E. coli* O157:H7, treatments that contained sodium fumarate (1 and 4) resulted in the most rapid inactivation, with a log reduction above the maximum reduction possible based on the detection limit of the method (6.07 log reduction) being observed for both treatments at 5 and 10 min CFU/mL (*p* < 0.05; Figure 5B).

When *S.* Typhimurium 30 was challenged with FS (pH 2.8) and HCL (pH 2.8) after 120 s, log reductions of 2.25 and 0.48 occurred, but these treatments were not found to offer significant reduction. With the six reformulated treatments 1, 2, 3, 4, 5 and 6 that were based on the original FS (pH 2.8), a 6.05, 3.78, 3.42, 6.22, 3.24 and 1.48 log reduction of CFU/mL occurred, respectively. In the case of *S.* Typhimurium, 30 treatments 1–5 were found to offer significant reductions after 120 s. However, only treatments 1 and 4 were found to offer significant improvement over the original FS treatment (pH 2.8), while these two treatments that contained sodium fumarate (1 and 4) also resulted in the most significant and rapid inactivations (*p* < 0.05; Figure 6). Inactivation in the presence of sodium fumarate was so high that even at 30 s, as numbers of *S.* Typhimurium 30 were below the detection limit (>6.1 log reduction of CFU/mL), while all other treatments had a lower than 2.5 log reduction of CFU/mL at that time point (Figure 6). Due to the centrifugation of the overnight cultures used, the detection limit varied slightly between replicates and samples, and this is why we reported the detection limit as >6.1.

## 4. Discussion

Disinfection is a very important process that reduces pathogens and spoilage microorganisms from fresh produce, enhancing food safety and sustainability. In previous work, we have shown that organic acid disinfectants do not select for multiple antimicrobial resistance [42,43,44]. Enhancing the efficacy of organic acid disinfectants could be achieved by using acids with low pKa, which normally possess higher antimicrobial activity. Furthermore, a lot of these weak acids have low toxicity, which is an important property for disinfectants.

Commonly, scientists aim to design harsher regimes to decontaminate fresh produce. However, we have previously demonstrated an alternative novel way by which we could increase the acid sensitivity of microorganisms by inhibiting amino acid decarboxylase systems, such as the GAD system, without affecting acidic conditions and the harshness of the treatment to the fresh produce [21]. One compound that can inhibit the GAD system of *L. monocytogenes* is fumaric acid [20]. Apart from *L. monocytogenes*, fumaric acid has been shown previously to be active against a wide range of other foodborne pathogens, including *E. coli* and *Salmonella* sp. [22,23,24,25,45,46]. The activity of fumarate against *E. coli* might have the same basis as with *L. monocytogenes* since it is also an inhibitor of the *E. coli* GAD system [47]. Currently, it is not known how fumarate acts on *Salmonella*, but it could be through a similar effect on the lysine, ornithine, arginine decarboxylase systems, or effects on metabolism since it is an important bacterial metabolite. There is also a possibility that fumaric acid could impact on these organisms through the disruption of metabolic functions, as it is part of the tricarboxylic acid cycle (TCA); however, further work is required to assess this.

Initial experiments were undertaken to establish the MICs of some key components of FS (tartaric, citric and malic), as well as those of fumaric acid and sodium fumarate. The MICs indicate that fumaric acid was not the most inhibitory of the acids tested, while the MIC of citric acid was 12.5 mM against *E. coli* O157:H7. Sodium fumarate did not possess an MIC at low concentrations (>200 mM) since it is effective only at acidic conditions [48] and as a salt, it does not reduce the pH of the environment [31]. However, fumarate is highly active at acidic conditions, and therefore, we investigated its use in acidic disinfection of fresh produce, which is a process aiming to inactivate a high number of microorganisms in a short period of time. From previous work, we know that fumarate can enhance the antimicrobial activity of the above process, while it is also active against biofilms of *L. monocytogenes* [20].

Therefore, the next step was to investigate the effect of sodium fumarate in vitro when combined with a commercial acidic disinfectant (FS). We did not use a cocktail of strains, as our aim was to see the behaviour of previously studied individual strains that are relevant to acid resistance to account for the worst-case scenario. The next step will be to use a cocktail of different strains. These experiments were performed with *L. monocytogenes* LO28, *E. coli* O157:H7 and *S.* Typhimurium 30. *L. monocytogenes* LO28 is a well-known typed strain that has been used in acid work previously, while it is relatively acid resistant in the TSBY medium as it is able to effectively utilise the GAD system, which is the main determinant of acid resistance in *L. monocytogenes* [34,49,50]. TSBY medium was used as it is of plant origin (soy) since we aimed to perform experiments in fruits (plant material). Furthermore, the choice of *E. coli* O157:H7 was relevant to this work as it is one of the most important pathogens found on fresh produce, while it has a significant acid-resistance, higher than that of other strains such as K-12 under complex environmental conditions [51]. *S*. Typhimurium 30 is also an important pathogen of faecal origin that can affect fresh produce, while it has also been previously used in work with acid resistance and disinfection [35].

Ten mM sodium fumarate increased the bactericidal effect of FS against the above 3 pathogenic bacteria (Figure 1A–C) [52,53]. Similar results were obtained with *L. monocytogenes* 10403S (Figure S1 Supplementary Data), *E. coli* K12 (Figure S2 Supplementary Data), indicating that sodium fumarate is active against 6 different strains from 4 species. This underpins a wide range of antimicrobial activity against various microorganisms, suggesting that it is a great candidate for use in disinfection.

Acid resistance comparison between all the 3 different species against sodium fumarate is not feasible, since different media were used in each case. However, we found that *E. coli* O157:H7 (Figure 1B) was more resistant than *S.* Typhimurium 30 (Figure 1C) under similar conditions. The type of medium is a significant parameter of acid resistance since it affects the expression and activity of amino acid decarboxylase systems, as previously shown for *E. coli* and *L. monocytogenes* [34,49,50].

Subsequently, we proceeded with experiments investigating the antimicrobial activity of FS supplemented with 10 mM sodium fumarate (pH 2.4) in fresh produce (strawberries, pears and apples). However, the latter combination did not offer any improvement in antimicrobial efficacy over the use of FS alone (data not shown). This limited antimicrobial activity of FS with 10 mM sodium fumarate on the bacteria may be explained by the complex nature of fresh produce, as bacteria may embed themselves on the irregular surface of fresh produce or bind to specific sites such as stomata [7,54]. Therefore, we proceeded with the use of FS supplemented with 25 mM sodium fumarate (pH 2.4), while other treatments with (a) water, (b) chlorine 100 ppm, (c) FS (pH 2.4) were included as controls. FS with 25 mM sodium fumarate at pH 2.4 was found to have a greater effect than FS alone at a similar pH of 2.4. On strawberries, apples and pears, the combined treatment was found to be significantly more effective than 100 ppm free chlorine and the original FS treatment (Figure 3 and Figure 4; *p* < 0.05).

We did not find any pattern in terms of inactivation where a specific microorganism was much more sensitive than the others. This is interesting as in the in vitro experiments in broth, *E. coli* O157:H7 was found to be significantly more resistant than *S.* Typhimurium 30 (Figure 1B,C). In addition, we observed that the inactivation of all three microorganisms elicited by the combined FS with 25 mM sodium fumarate at pH 2.4 was less prominent in strawberries than in the other two fruits (Figure 2, Figure 3 and Figure 4). Previously, it has been shown that decontamination of strawberries is less efficient due to their rough surface [55]. The results presented here demonstrate that a typical sodium hypochlorite treatment (100 ppm) and a commercial organic acid treatment at pH 2.4 showed limited effects (Figure 2, Figure 3 and Figure 4), which have also been noted previously [15], while sodium fumarate offers a significant advantage.

To further improve the FS treatment, we prepared 6 different mixes of organic acids, containing key components of FS (4.4 mM tartaric acid, 14.5 mM monosodium citrate, malic acid), while 2 contained sodium fumarate (mixes detailed in Table 2). In treatment 1, 50 mM sodium fumarate substituted 14.5 mM monosodium citrate, while in treatment 4, 25 mM sodium fumarate substituted 4.4 mM tartaric acid. Overall, for *L. monocytogenes* LO28, the two treatments containing sodium fumarate (1 and 4) were superior among all others, achieving a significant >6 log reduction of CFU mL^−1^ at 5, 10 and 20 min (*p* < 0.05; Figure 5A). This suggests that these treatments are more effective at removing *L. monocytogenes* LO28 than FS, indicating the enhanced antimicrobial activity of fumarate. When *E. coli* O157:H7 was challenged with the different treatments of mixed acids, 1 and 4 offered significant improvements over the original FS treatment at 20 min, with treatment 1 offering the most rapid and superior inactivation than all other treatments at 5 and 10 min (*p* < 0.05; Figure 5B). All other treatments showed a similar performance to the original FS. In the experiments with *S*. Typhimurium 30, a shorter time scale was utilised in initial experiments because this organism was sensitive to the treatments compared to the other two bacteria used. Interestingly, this significant difference in sensitivity between *S*. Typhimurium 30 and the other two bacteria was not observed in the experiments with fruits, supporting the idea that the type of fruit was a more significant parameter in the efficiency of the disinfection regime than the type of microorganism. In the case of *S*. Typhimurium 30, over this shorter time scale, treatments 1, 2 and 4 proved to be more effective than the original FS treatment against *S*. Typhimurium (*p* < 0.05; Figure 6). Treatments 1 and 4 had the citric acid used in the original FS treatment replaced with trisodium citrate, while they had 50 mM and 25 mM sodium fumarate added, respectively. Clearly, the critical factor enhancing antimicrobial activity in treatments 1 and 4 was the sodium fumarate, since the trisodium citrate contained in treatments 5 and 6 did not confer any major antimicrobial effect. Based on the above, sodium fumarate shows a high degree of antimicrobial activity under acidic conditions, and it could be used to improve the effectiveness of acidic disinfectant treatments for fresh produce. However, in relatively higher concentrations, such as 50 mM, it has been shown to cause a browning effect on some types of produce [22,24,45].

These results highlight a significant improvement of acidic disinfection regimes, with the addition of a relatively low concentration of 25 mM sodium fumarate. Furthermore, with the increased trend of consumption of fresh produce, which has been associated with increased incidence of foodborne illness, the need for more effective disinfection is stronger than ever [15].

Most countries around the world, including the USA (FDA), permit the use of sodium fumarate (E365) as a food additive, while the EU limits its use as it is allocated a low numerical ADI of 6 mg/kg by the EU scientific committee [27,28]. A similar ADI is allocated for fumaric acid (E297), but due to its low solubility (<0.7% *w*/*v* or 60 mM) [41] in aqueous solutions, its use is permitted in various applications in the EU [29] although it is currently under re-evaluation by EFSA [30]. However, the current work does not investigate the use of sodium fumarate as an additive, but as a disinfectant and based on the above encouraging results and its high solubility of 22% (easier usage), it could be considered for this specific application. With an ADI of 6 mg/kg, an average human of 70 kg should not consume more than 0.42 g sodium fumarate per day. The above concentration of 25 mM used corresponds to 0.40% sodium fumarate in the disinfection liquid, which by itself is far below the ADI. Following a treatment with the above disinfection liquid containing 0.40% fumarate and washing, this concentration will be reduced dramatically by a factor of 100–1000, resulting in 0.004–0.0004% in the washing water and eventually leaving minimal traces on the fruit. This would be significantly far below the maximum permitted levels. For example, if we assume that sodium hypochlorite (ADI: 0.15 mg/kg) and sodium fumarate (ADI: 6 mg/kg) are roughly diluted and removed in a similar rate during the disinfection process, and used 25 mM (4 g/L) sodium fumarate or the maximum allowed of 200 ppm (0.2 g/L) for sodium hypochlorite, then we could divide the above used concentrations by the ADI for each substance and find out how much of the substance we use compared to its ADI. With the above calculation, we find that the ratio of (max used concentration)/ADI is 1333.33 for sodium hypochlorite and 666.66 for sodium fumarate. This means that we allow more than double the concentration of sodium hypochlorite than the set concentration for sodium fumarate.

Our work here also shows the beneficial effects of the usage of sodium fumarate in combination with other organic acids in real-life disinfection scenarios of fresh produce. Even the presence of low concentrations of sodium fumarate (25 mM) could increase the bactericidal effects of a current commercial treatment on a range of fresh produce such as strawberries, apples and pears. It is possible that such a treatment could be used in these products in the future. Furthermore, it is also clear that organic acid washes, especially when optimised as shown here, can provide a rapid and effective disinfection compared to more conventional treatments, such as chlorine washes. In addition, through formulating more effective treatments, such as those presented here with sodium fumarate, it may be possible to achieve effective decontamination at higher pH, reducing the impact of such treatments upon the environment and upon the produce. As such, we could reduce foodborne illness, reduce spoilage and thus increase sustainability.

## 5. Conclusions

Sodium fumarate inhibits the GAD system of *L. monocytogenes*, *E. coli* and possibly many more bacteria. As such, this is a novel concept of not focusing on enhancing the harshness of disinfectant, but in contrast to inhibiting the main bacterial acid resistance mechanisms as a means to enhance its antimicrobial activity. We show here that sodium fumarate can enhance the antimicrobial activity of a commercial acidic disinfectant in broth cultures of *L. monocytogenes*, *E. coli*, *S.* Typhimurium. Furthermore, these effects were applicable in strawberries, apples and pears for the first 3 bacterial species. Future work should focus on capitalising on this effect, optimising it and ensuring the improvement of disinfection regimes for fresh produce, aiming to reduce foodborne illness worldwide.

## Figures and Tables

**Table 1:** List of bacterial strains examined in this study.

Strains	Relevant Properties	Source
*L. monocytogenes* LO28	Serotype 1/2c, WT clinical isolate from human faeces	Cotter et al. (2001) [36]
*E. coli* O157:H7	Non verocytotoxic strain not possessing either *stx1* or *stx2* shiga toxin genes (Woodward et al., 2003) [37]	Central Public Health Laboratory, London. National Culture Type Collection (NCTC)12900
*S.* Typhimurium 30	Designation DT104 strain 30 from bovine source (Payne et al., 1992) [38]	Jørgensen et al. (2000) [39]

**Table 2:** Composition of weak acids for each treatment applied against *L. monocytogenes* LO28, *E. coli* O157:H7 and *S.* Typhimurium 30.

Weak Acid (mM)	Treatment					
	1	2	3	4	5	6
Tartaric acid	4.4	4.4	4.4		4.4	4.4
Trisodium citrate	14.5			14.5	14.5	14.5
Citric acid anhydrous		14.5	14.5			
Malic acid	14.6	64.6	39.6	14.6	64.6	39.6
Sodium fumarate	50			25		
Total acid anions	69	83.5	58.5	54.1	83.5	58.5

**Table 3:** MICs in mM observed for the organic acids tested against *L. monocytogenes*, *E. coli* and *S.* Typhimurium.

Organic Acid & pKa Values	MIC (mM)		
	*L. monocytogenes* LO28 WT	*E. coli* O157:H7	*S.* Typhimurium 30
Tartaric acid (pK_a1_ = 2.98\pK_a2_ = 4.30)	50	25	25
Citric acid (pK_a1_ = 3.13\pK_a2_ = 4.76\pK_a2_ = 6.40)	50	12.5	25
Malic acid (pK_a1_ = 3.40\pK_a2_ = 5.11)	50	25	50
Fumaric acid (pK_a1_ = 3.02\pK_a2v_ = 4.38)	34	34	34
Sodium fumarate (pK_a1_ = 3.02\pK_a2_ = 4.38)	>200	>200	>200

## Data Availability

The original contributions presented in this study are included in the article/Supplementary Materials. Further inquiries can be directed to the corresponding author.

## Supplementary Materials

The following supporting information can be downloaded at: https://www.mdpi.com/article/10.3390/foods15132339/s1, Figure S1: Survival of *L. monocytogenes* 10403S in the presence of FS alone (◇) or FS supplemented with 10 mM sodium fumarate (◆) at pH 2.8 (A) or pH 2.4 (B). Asterisks denote statistical significance using a paired student *t*-Test (*p* < 0.05), while D.L denotes detection limit of the experimental setup; Figure S2: Survival of *E. coli* K-12 in the presence of FS alone (△) or FS supplemented with 10 mM sodium fumarate (▲). The pH of all challenges was 2.4 while asterisks denote statistical significance using a paired student *t*-Test (*p* < 0.05), and D.L denotes detection limit of the experimental setup).
