# Supplementary material for: Sodium Fumarate Enhances the Antimicrobial Efficacy of a Commercial Acidic Disinfectant Against Listeria monocytogenes, Escherichia coli and Salmonella Typhimurium Inoculated on Fresh Produce
Source: Foods. 2026 Jul 2;15(13):2339. doi: 10.3390/foods15132339 (PMC13361480; doi:10.3390/foods15132339)
Supplement: Supplementary file 1 [file foods-15-02339-s001.zip › foods-4235378-supplementary.pdf]

Supplementary data

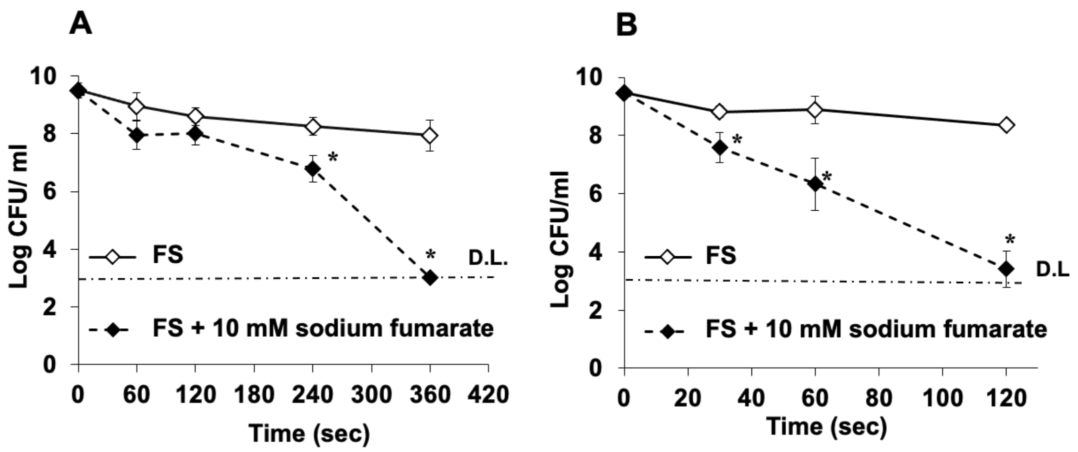

Figure S1. Survival of *L. monocytogenes* 10403S in the presence of FS alone (◇) or FS supplemented with 10 mM sodium fumarate (◆) at pH 2.8 **(A)** or pH 2.4 **(B)**. Asterisks denote statistical significance using a paired student T-Test (P < 0.05), while D.L denotes detection limit of the experimental setup.

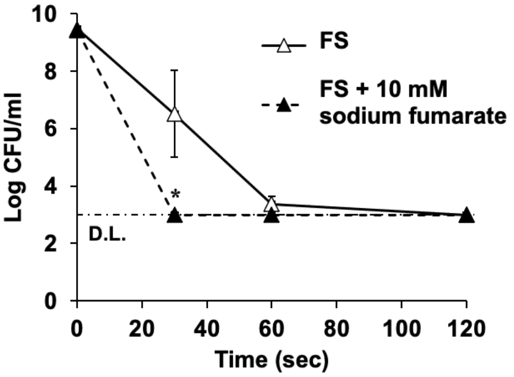

Figure S2. Survival of *E. coli* K-12 in the presence of FS alone (△) or FS supplemented with 10 mM sodium fumarate (▲). The pH of all challenges was 2.4 while asterisks denote statistical significance using a paired student T -Test (P < 0.05), and D.L denotes detection limit of the experimental setup).
